# Influenza Vaccination Experiences of Pregnant Women as a Predictor of the Intention to Become Vaccinated in Future Pregnancies in Spain

**DOI:** 10.3390/vaccines8020291

**Published:** 2020-06-09

**Authors:** Noelia Rodríguez-Blanco, José Tuells, Andreu Nolasco

**Affiliations:** 1 Department of Obstetrics and Gynecology, Hospital Universitario del Vinalopó, Spain C/Tonico Sansano Mora, 14, 03293 Elche, Spain; noelia.rodriguez@uchceu.es; 2 Department of Nursing Universidad CEU Cardenal Herrera, Plaza Reyes Católicos, 19, 03204 Elche, Spain; 3 Department of Community Nursing, Preventive Medicine and Public Health and History of Science, University of Alicante, San Vicente del Raspeig, 03690 Alicante, Spain; nolasco@ua.es

**Keywords:** influenza, pregnant women, vaccine uptake, maternal vaccination, childhood vaccination, vaccines acceptance

## Abstract

A good perception of the vaccines administered during pregnancy favors immunization coverage, which is still not optimal for the influenza vaccine. To understand the predisposition towards vaccination in future pregnancies, a study was performed that evaluated the experiences of women with the vaccine or influenza. A cross-sectional study was conducted through telephone interviews given to a total of 683 postpartum women in two health departments from the Valencia Community (Spain). This interview asked about their intention of becoming vaccinated in future pregnancies and whether they were favor or against vaccination. Most of them, 98.5% (*n* = 673 [95% CI: 97.6–99.4]) (*p* < 0.001) declared having received the systematic vaccines throughout their lives. The ones who were vaccinated against influenza, 91.9% (*n* = 387 [95% CI: 89.2–94.6]) (*p* < 0.001) manifested they would do so in future pregnancies. The probability of future non-vaccination was modeled, which was related to an unfavorable opinion towards vaccines (OR = 4.07 [95% CI: 2.01–8.24]) (*p* < 0.001), having suffered from influenza during pregnancy (OR = 3.84 [95% CI: 1.41–10.42]) (*p* < 0.05), and not having been vaccinated during previous pregnancies (OR = 38.47 [95% CI: 23.58–62.76]) (*p* < 0.001). Vaccination during pregnancy increases the intent of vaccination in the future.

## 1. Introduction

Vaccination of pregnant women (“maternal vaccination”), has greatly increased in the 21st century. The protection received by the mother and fetus in a single vaccination implies benefits both for the mother, who is protected from infection and its complications, as well as the fetus and newborn, due to the transfer of antibodies [1,2].

This is why, it is important to know and understand the predictors or factors that affect the acceptance of maternal vaccines [3], as well as to evaluate the mother–fetus repercussions after the immunization itself, as women can have multiple pregnancies and therefore various options for vaccination [2,4].

To increase the probability of living in a healthy perinatal state, it is important to provide pre-conception care that assesses the exposure to infectious diseases, explores the immune status of the future mother, and administers the vaccines that could be required [5]. Likewise, a greater protection of the mother–child pairing can be achieved through the use of vaccines during the pregnancy itself, with the safest ones being the ones composed by dead or inactive viruses or bacteria or polysaccharide ones [2]. After birth, a new opportunity for vaccination arises, which can provide immunity to the mother as an anticipation of future pregnancies [6]. Any of these three points in time is an excellent occasion for increasing the mother’s knowledge of childhood vaccines and for promoting a favorable predisposition towards immunization programs [7,8].

Thus, in the field of women’s health, maternal vaccination has become a new opportunity for public, obstetric, and pediatric health, signifying modern healthcare in which the mother can transfer antibodies, thus providing protection to the fetus, to the newborn and the pregnant mother as well [9].

Currently, two vaccines are recommended during pregnancy, influenza and Tdap (Tetanus, Diphtheria, Pertussis for adults), but in the near future, new possibilities could arise for including new vaccines (i.e., respiratory syncytial virus, herpes simplex virus, and cytomegalovirus vaccines.) [10]. Faced with the new option of adding new vaccines during pregnancy, it is interesting to know and assess the experiences of women after receiving vaccines and how they perceived the repercussions on their health and the child’s as well [4]. A key element for the success of a vaccination program is transmitting the future mothers a good opinion about the safety and efficiency of the vaccines, which are the most influential factors when deciding to vaccinate or not [11]. Fortunately, the vaccines recommended against influenza and whooping cough have a good safety profile [2,12]. However, although the WHO has recommended the addition of the influenza vaccine for pregnant women in national programs of immunization in 2012 [13], the desired coverage has not been reached, and vaccination rates are still low [14,15].

In Spain, as in other countries, the coverage against influenza is low and different in each of the 17 autonomous communities. Furthermore, there is no single national vaccination registry, as the system is decentralized and each autonomous community has its own registry [16,17,18]. In the Valencian Community, the declaration and registration of vaccines are carried out electronically through the Nominal Vaccine Registry (NVR) [19].

Given that women can become pregnant many times during their reproductive cycle, studying their predisposition for receiving or not the influenza vaccine during each pregnancy, as well as the factors that determine their decision, will allow detecting elements that could be improved upon in the coverage of the influenza vaccine. Previous research studies have evaluated the barriers or reasons for not being vaccinated against influenza [20,21], but not many studies have been found that aimed at assessing the intention of the pregnant women to become vaccinated in the future after their previous experience with the influenza vaccine or having suffered from the illness [4]. Different studies have observed that the advice and recommendations provided by a health professional during pregnancy are key for achieving a good vaccination coverage [4,11].

The objective of this research was to discover the intent of postpartum women to be vaccinated or not against influenza in a future pregnancy, and the factors associated to this attitude after their previous experience with the vaccine or the illness, with a sample obtained from two health departments, Torrevieja (TV) and Elche-Crevillente (EC) from the Valencia Community (VC, Valencia, Spain).

## 2. Materials and Methods

### 2.1. Study Population

A cross-sectional study was conducted through a telephone interview with women who had given birth in the two university hospitals located in the health departments of TV and EC in the VC, Spain. Both hospitals have an average of 2600 births per year. The vaccination rate was 35% in the 2015–2016 flu vaccination season for pregnant women in the VC.

According to the VC protocol, all pregnant women are supervised and monitored by a midwife. From 19 October 2015, until 31 January 2016, the midwives provided flu vaccine advice to pregnant women and asked for their consent to participate in a telephone interview during the postpartum period, which was conducted six months after the end of the influenza season. The women who did not want to participate, were not localized on the phone after three calls, and those who had suffered a fetal loss were excluded from the study.

A minimum number of 549 pregnant women was estimated after calculating the sample size expected for estimating the vaccine coverage ratio with a confidence interval of 95%, an accuracy of 5%, and a loss to follow-up of 30%.

### 2.2. Measurements

During the initial visit with the midwife, the sociodemographic and obstetric variables of the pregnant women were recorded. The sociodemographic variables were health department (TV/EC), country of origin (Spain/Not Spain), age of the pregnant woman (years). The obstetric variables were previous pregnancies (0, ≥1), previous abortions (0, ≥1), and pregnancy weeks grouped as trimesters (T): 1 T (1–13 weeks), 2 T (14–27 weeks) and 3 T (28–40 weeks).

Their attitudes towards and experiences with flu vaccinations were obtained through a structured questionnaire designed *ad hoc*, through a telephone interview given to women who were in their postpartum period, ranging from 1 August 2016 to 30 October 2016. The questionnaire contained questions related to (1) the beliefs and opinions about vaccination in general; (2) a self-declaration of flu vaccination during pregnancy (vaccination coverage). Their answers were verified and corroborated by consulting the vaccination registry (NVR); (3) their reasons for rejecting the vaccine, for those who were not vaccinated, and the secondary effects for those who were vaccinated; (4) the attitude and predisposition for being vaccinated in a future pregnancy; (5) their own experience with the illness and/or the influenza vaccine throughout their lives.

### 2.3. Statistical Analysis

For all the variables, the frequencies and percentages of their categories were calculated, as well as the confidence intervals at 95% (95% CI) with the Chi-squared test to analyze the significance of the association between categorical variables. In the case of the quantitative categories, the mean and standard deviation were calculated.

The adjusted associations between variables and “country of origin”, “vaccination during the present pregnancy”, “beliefs or opinion about vaccines” and “experience with the flu illness”, with the future intent of non-vaccination, were estimated with the odds ratio (OR) and a 95% CI calculated with a multivariate logistic regression, utilizing the non-vaccination as the response variable and the rest as the explanatory variables.

All the analyses were performed with the SPSS program version 20.0. The level of significance was set at 0.05. For the multivariate logistic regression, a level of 0.10 was considered for remaining in the model.

### 2.4. Ethics Approval

The interviewed women were ensured about the confidentiality and anonymity of the data collected, as well as their right to not answer the questions. The study considered the ethical principles for medical research established in the current legislation. It was approved by the Research Ethics and Research Committee of the University Hospitals of Torrevieja and Elche-Crevillente on 10/26/15 and by the Spanish Agency of Medicine and Medical Products (AEMPS) (#134-14).

## 3. Results

### 3.1. Sociodemographic and Obstetric Characteristics of the Interviewees

The sample of postpartum interviewees totaled 683 individuals. Of these, 60.9% (*n* = 416) belonged to the EC health department and the rest to the TV one. Most of them, 77.6% (*n* = 530) had been born in Spain, and their average age was 31.4, with a standard deviation of 4.8. The obstetric value results showed that 95.2% of the interviewees had been pregnant before, 1.9% had suffered an abortion, and all of them had received vaccination advice from a midwife (28.0% on the first trimester of the pregnancy, 35.6% on the second, and 36.4% on the third) (Table 1).

### 3.2. Vaccination Self-Declaration and Concordance with the Registry (NVR)

The “previous vaccination during pregnancy” statement yielded a 61.6% flu vaccination coverage (*n* = 421 [95% CI: 56.9–66.2]). (Table 1).

The answers provided by the women in their postpartum period about their flu vaccination or not was compared with the existing data found in the Vaccination Registry of the Valencian Community (NVR) to verify the veracity of their responses. The results showed that for 98.8% (*n* = 655 [95% CI: 98.0–99.6]) of women, the statement coincided with the data available in the NVR records, indicating a high agreement and a high degree of sincerity in their responses.

### 3.3. Beliefs and Opinions about Vaccination in General

As observed in Table 2, the majority opinion of the pregnant women about the vaccines was favorable or very favorable in both groups (flu vaccinated and unvaccinated). This good opinion was more common for those who had previously received the vaccine. Only 0.6% (4/683) manifested their rejection of the vaccines. Also, 98.5% (*n* = 673 [95% CI: 97.6–99.4]) confirmed having received the systematic vaccines scheduled in the children’s calendar.

### 3.4. Intention towards Future Vaccination

Women who were vaccinated or unvaccinated during their recent pregnancy were asked if they would be vaccinated again against influenza in a future pregnancy, if it coincided with the period of the seasonal influenza campaign. Of the total women interviewed, 66.0% (*n* = 451 [95% CI: 61.6–70.4]) declared that they would be vaccinated again.

A significant difference in this intention was observed, which was greater for those who had been vaccinated in their last pregnancy, 91.9% (*n* = 387 [95% CI: 89.2–94.6]) as compared to those who had not been vaccinated, 24.4% (*n* = 64 [95% CI: 13.9–34.9]). (Table 2).

**Figure 1:**
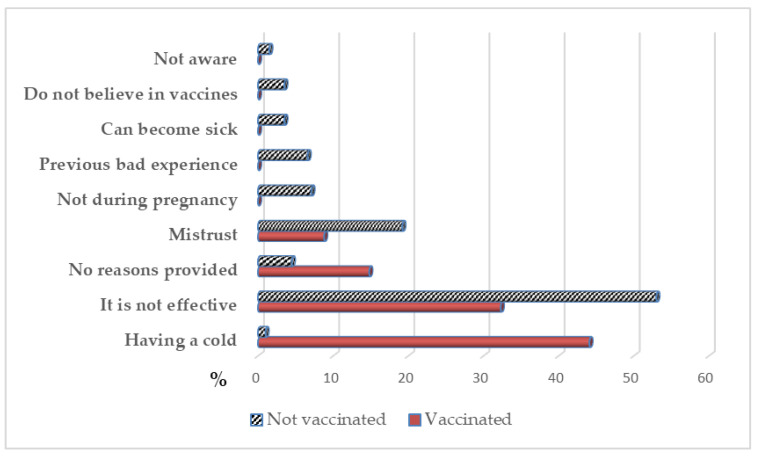
Reasons for rejecting vaccine in the next pregnancy.

The reasons provided for not wanting to become vaccinated in a future pregnancy can be observed in Figure 1. The main reason for not wanting to be vaccinated, provided by 50% of the pregnant women (vaccinated and unvaccinated) was the belief that the influenza vaccine “is not necessary or effective”. In second place, the women cited “the distrust” towards the vaccine (17.7%). Other reasons were “having a cold” when the vaccine was due (7.3%), “not wanting to” receive it while pregnant (6.0%), the “possibility of becoming sick” after receiving it, or “not believing in vaccines” (both 3.0%). Only 1.3% of the sample stated “not knowing the recommendations” for becoming vaccinated against influenza during pregnancy.

### 3.5. Secondary Effects after the Vaccination

Of the 61.6% (*n* = 421 [95% CI: 56.9–66.2]) of pregnant women who received the vaccine against influenza, 8.6% (*n* = 36 [95% CI: 0.0–17.7]) manifested suffering secondary effects after the vaccination.

The secondary effects experienced were: symptoms of a cold without fever, 47.2% (*n* = 17 [95% CI: 23.5–70.9]), pain and inflammation on the arm where they received the injection, 33.3% (*n* = 12 [95% CI: 6.6–59.9]) and “becoming sick” with influenza after immunization, 16.7% (*n* = 6 [95% CI: 0.0–46.5]). There was a case of hospital admittance due to the risk of premature birth post-vaccine.

For 44.1% (*n* = 15 [95% CI: 19.0–69.2]) of the vaccinated pregnant women, suffering a “cold” after vaccination was the main reason for not wanting the vaccine in later pregnancies. However, in the unvaccinated group, the belief that the vaccine was not “necessary or effective” was the main reason for rejection, 53.0% *n* = 105 [95% CI: 43.4–62.5]) (Figure 1).

### 3.6. Personal Experience Related with the Flu

The personal experience with influenza throughout their life was also evaluated. Most of the interviewees had never contracted influenza, representing 73.5% (*n* = 502 [95% CI: 69.6–77.4]) of the total. Among the unvaccinated group, this ascended to 79.3% *n* = 208 [95% CI: 73.8–84.8]) (Table 2).

Having been sick from influenza in the past, while not pregnant, was a predisposing factor for becoming vaccinated during the present pregnancy (*p* < 0.05). Only 5.3% affirmed having suffered influenza during another pregnancy. This subgroup was divided similarly between vaccinated and unvaccinated women.

### 3.7. Multivariate Analysis with the Future Intent of Non-Vaccination as the Response Variable

The results obtained after adjusting the multivariate logistic regression models to explain the future intent of non-vaccination against influenza can be observed in Table 3.

The probability of non-vaccination was modeled, resulting in the significant association with having an unfavorable opinion about vaccines in general (*p* < 0.001), (OR = 4.07 [95% CI: 2.01–8.24]), having experienced influenza during pregnancy (*p* < 0.05), (OR = 3.84 [95% CI: 1.41–10.42]), to not having been previously vaccinated against influenza during pregnancy (*p* < 0.001), (OR = 38.47 [95% CI:23.58–62.76]), and lastly, that the pregnant woman was of Spanish origin (*p* = 0.082), (OR = 1.65 [95% CI: 0.93–2.91]).

## 4. Discussion

In general, mothers have an excellent opinion about the vaccines received during their childhood. This is reflected in the good childhood vaccination coverage that is traditionally found in Spain [17], and is certified by the high percentage of women who ensured having received all the vaccines throughout their lives. The adult women, therefore, were vaccinated in their childhood and will now have to make decisions about maternal immunization in this prenatal phase, and the future immunization of their children as well [22]. And, this is a key aspect for achieving a similar coverage reached with the childhood vaccination calendar [10].

The objective of this study was to discover if the previous life experiences with the influenza vaccine or related to influenza, could affect or have an influence on the future decision to vaccinate. In the present work, having been vaccinated against influenza in their last pregnancy was a factor associated with a greater acceptance of vaccination, as in other studies [4,23,24] Two-thirds of the women polled (66%) would again vaccinate against influenza in future pregnancies, a percentage that is higher than the one obtained (39%) by van Lier et. al. [25].

The women’s personal experience with influenza throughout their life, not having experienced it or having experienced it during pregnancy or while not pregnant, were other subjects of interest. As a result, it was found that although a small percentage (5.3%) had been sick with influenza while pregnant, this factor was significantly associated with the future intent of non-vaccination. This data should be clarified, as many pregnant women defined influenza as having had a cold or another respiratory infection, without having a serological diagnosis that could confirm its presence. In this sense, although the secondary effects described by the postpartum women after the vaccination were few (9.1%) and without severity [26], almost half of the effects declared were related to having suffered a cold “without fever” after the vaccination. Many pregnant women believed that the influenza vaccine could cause the infection itself and mentioned cold-like symptoms and processes “without fever” that they defined as influenza [27]. This erroneous belief could imply a barrier for the future vaccination of women.

In Europe, to achieve a vaccination coverage against the flu higher than 75% [18], it is important for women to attend prenatal visits or follow a pregnancy monitoring program [28]. In our study, we obtained a vaccination coverage close to 62.0%, a figure that was higher than that declared at the regional and state levels (lower than 34.0%) [19,29]. Also, the vaccination self-declaration obtained a high level of agreement with the NVR of the VC, which confirms the frankness of the postpartum women during the interview.

The pregnant women who were not native to Spain were vaccinated against the flu more often than Spanish women, as opposed to a study conducted in France, where immigrant women were considered a risk factor for non-vaccination [30]. Our women sample accepted vaccination in more homogeneously in any trimester of pregnancy, as opposed to the results from a study by Groom et. al [24].

The flu vaccination recommendation was directly provided by the midwife during the consultation [31,32]. We believe that a professional health worker with knowledge and skills obtains significantly higher vaccination coverage. Thus, in a research study [33] such as the present one, after a specific training program for midwives, the probabilities of the women who received the flu vaccine during pregnancy were significantly greater after the implementation of the program. The midwives were able to establish a relationship of trust, availability, and support during the decision-making process of the pregnant women related their health and that of their future child, and also performed promotion and mother—child education activities as part of their habitual activities [34,35,36] On the other hand, the midwives asked for more time, resources and continuous training [37,38,39]. The recommendation of the professional health worker, having the vaccine available and free in the consultation itself, were favorable factors as observed in our study [28,40].

If we combine the good and majority opinion of the pregnant women towards vaccines in general with the favorable attitude of the health professionals, then expectations for improvement of the forecasted future vaccination coverage for the child calendar are high [41]. The reticence or opposition towards the vaccines were not considered a problem in the present research study, where less than 4% recognized not being in favor, in line with other studies [40]. However, the small number of pregnant women who were against vaccination expressed their rejection of the flu vaccine in future pregnancies. In this study, the secondary effects after vaccination were slight and small in number, with the main reason for not vaccinating again being related to having suffered from a cold after the vaccination.

As for the flu vaccine, we received responses such as “it is not an effective or necessary vaccine” and “I have doubts or mistrust about its safety”, which represented more than half of the reasons provided for rejecting it in future occasions [27,32,42]. Pregnant women differ from those who are not, because they have concerns about the safety of vaccines for the fetus, and some prefer not to become vaccinated during pregnancy [11]. Key aspects for improving the low flu vaccine coverage of the pregnant women include knowing the reasons provided by the women who rejected the vaccine, having health professionals who are trained to provide answers to their doubts, provide more opportunities for vaccination and include prenatal vaccine recommendations, creating a vaccine history for every woman [32]. Professional monitoring after immunization is an influencing factor in future maternal vaccinations, also considering the repetition of the doses or the increase in the number of new vaccines in this period of life of the women [27].

It would be advisable to create trust in vaccines during pregnancy. The midwives, together with the obstetricians and pediatricians, could collaborate in the creation of training programs related to maternal and child vaccination before the birth [43].

More research studies on this subject could result in the development of future recommendations about the optimal period of time for immunization, (pre-conception, during pregnancy or postpartum) and the most cost-effective professionals, not only in the vaccination, but also in the monitoring and advice phases [44,45].

As a limitation of the study, it should remembered that the women indicated having fallen ill with influenza in the past or had this disease as a side effect after vaccination, according to her own experience. The vaccination status was also self-reported, and could therefore be subject to recall biases. However, this statement obtained a high level of agreement with the computerized nominal registry of vaccines from the VC (NVR).

## 5. Conclusions

The experience of influenza vaccination of pregnant women tends to condition future vaccination events. Given the vaccine is usually administered on various occasions, if some reason existed related to the secondary effects that the pregnant women attributed to the vaccine, it could lead to its rejection. The collaborative work by the health professionals involved in pregnancy care should take advantage of the good perception the women have about vaccines to provide efficient advice that will increase vaccination coverage.

## Figures and Tables

**Table 1:** Frequencies *n*, percentages % distribution of the women interviewed.

		Influenza		Influenza		Total		p *
		Vaccinated		Unvaccinated				
		*n*	%	*n*	%	*n*	%	
Department	TV	189	70.8	78	29.2	267	39.1	0.001
	EC	232	55.8	184	44.2	416	60.9	
Country of Origin	Spain	322	60.8	208	39.2	530	77.6	0.397
	Not Spain	99	64.7	54	35.3	153	22.4	
Previous pregnancies	1	11	39.3	17	60.7	28	40.1	0.009
	2	406	62.9	239	37.1	645	94.4	
	≥3	4	40.0	6	60.0	10	1.5	
Abortion	No	415	61.9	255	38.1	670	98.1	0.100
	Yes	6	46.2	7	53.8	13	1.9	
Parity	0	14	42.4	19	57.6	33	4.8	0.160
	≥1	407	62.6	243	37.4	650	95.2	
Trimester of the pregnancy	1°	118	61.8	73	38.2	191	28.0	0.220
	2°	159	65.4	84	34.6	243	35.6	
	3°	144	57.8	105	42.2	249	36.4	
Vaccination coverage		421	61.6	262	38.4	683	100	NA
Age	Mean (SD)	31.1	(5.6)	31.8	(4.8)			

* *p*-values of Chi-square test to check the significance of the differences among categories. TV = Torrevieja; EC = Elche-Crevillente; SD = Standard Deviation; NA = No analysis.

**Table 2:** Opinion on vaccines. Attitudes and experience related to influenza vaccination.

	Vaccinated	Unvaccinated	Total	CI 95%
	*n* = 421 (%)	*n* = 262 (%)	*n* = 683 (%)	
In general, your opinion about vaccines as a whole is? *				
Very favorable	92 (21.9)	35 (13.4)	127 (18,6)	[11.8–25.3]
Favorable	301 (71.5)	173 (66.0)	474 (69.4)	[65.2–73.5]
Indifferent	21 (5.0)	36 (13.7)	57 (8.3)	[1.1–15.4]
Unfavorable	7 (1.7)	14 (5.3)	21 (3.1)	[0.0–10.5]
Against	0 (0.0)	4 (1.5)	4 (0.6)	[0.0–8.1]
Throughout your life, have your received the vaccines scheduled in the vaccination calendar? *				
Yes	417 (99.0)	256 (97.7)	673 (98.5)	[97.6–99.4]
No	4 (1.0)	6 (2.3)	10 (1.5)	[0.0–9.3]
Would you get a flu shot if you became pregnant again? *				
Yes	387 (91.9)	64 (24.4)	451 (66.0)	[61.6–70.3]
No	34 (8.1)	198 (75.6)	232 (34.0)	[27.9–40.1]
**Have you suffered from the flu during pregnancy or while not pregnant?**				
Yes, pregnant	23 (5.5)	13 (4.9)	36 (5.3)	[0.0–12.6]
Yes, not pregnant *	106 (25.2)	43 (16.4)	149 (21.8)	[15.2–28.5]
Never	294 (69.8)	208 (79.3)	502 (73.5)	[70.0–77.7]

* Statistically significant differences between vaccinated and non-vaccinated (*p* < 0.001). CI: Confidence Interval.

**Table 3:** Multivariate logistic regression analysis to estimate the association and predictive ability about the predisposition for the future intent of non-vaccination.

Variables	*p*	OR	CI 95%
**Opinion about vaccines** Unfavorable Favorable	<0.001	4.07 1.00	(2.01–8.24)
**Have you suffered from influenza previously?** Yes, Pregnant Yes, not Pregnant Never	0.029	3.84 1.03 1.00	(1.42–10.42) (0.58–1.83)
**Have you been vaccinated previously?** No Yes	<0.001	38.4 1.00	(23.58–62.77)
**Country of origin** Spanish Not Spanish	0.082	1.65 1.00	(0.94–2.92)

CI: Confidence Interval.
